# Targeting Metabolic–Redox Nexus to Regulate Drug Resistance: From Mechanism to Tumor Therapy

**DOI:** 10.3390/antiox13070828

**Published:** 2024-07-10

**Authors:** Yuke Wang, Jingqiu He, Shan Lian, Yan Zeng, Sheng He, Jue Xu, Li Luo, Wenyong Yang, Jingwen Jiang

**Affiliations:** 1 West China School of Public Health and West China Fourth Hospital, West China School of Basic Medical Sciences & Forensic Medicine, Sichuan University, Chengdu 610041, China; wangyuke0213@163.com (Y.W.); hejingqiu@stu.scu.edu.cn (J.H.); lianshan1@stu.scu.edu.cn (S.L.); zengyan9908@163.com (Y.Z.); hesheng085@163.com (S.H.); xujuejoyce@163.com (J.X.); 2 Center for Reproductive Medicine, Department of Gynecology and Obstetrics, West China Second University Hospital, Sichuan University, Chengdu 610041, China; luoli0812@126.com; 3 Key Laboratory of Birth Defects and Related Diseases of Women and Children (Sichuan University), Ministry of Education, Chengdu 610041, China; 4 Department of Neurosurgery, Medical Research Center, The Third People’s Hospital of Chengdu, The Affiliated Hospital of Southwest Jiaotong University, The Second Chengdu Hospital Affiliated to Chong-Qing Medical University, Chengdu 610041, China

**Keywords:** metabolism, ROS, redox modification, drug resistance, dietary interventions

## Abstract

Drug resistance is currently one of the biggest challenges in cancer treatment. With the deepening understanding of drug resistance, various mechanisms have been revealed, including metabolic reprogramming and alterations of redox balance. Notably, metabolic reprogramming mediates the survival of tumor cells in harsh environments, thereby promoting the development of drug resistance. In addition, the changes during metabolic pattern shift trigger reactive oxygen species (ROS) production, which in turn regulates cellular metabolism, DNA repair, cell death, and drug metabolism in direct or indirect ways to influence the sensitivity of tumors to therapies. Therefore, the intersection of metabolism and ROS profoundly affects tumor drug resistance, and clarifying the entangled mechanisms may be beneficial for developing drugs and treatment methods to thwart drug resistance. In this review, we will summarize the regulatory mechanism of redox and metabolism on tumor drug resistance and highlight recent therapeutic strategies targeting metabolic–redox circuits, including dietary interventions, novel chemosynthetic drugs, drug combination regimens, and novel drug delivery systems.

## 1. Introduction

Drug resistance is the main cause for the failure of chemotherapy and targeted therapy in cancer treatment. Based on the underlying mechanisms, drug resistance can be categorized into primary and acquired resistance [1]. The specific mechanisms include increased drug efflux, alterations in drug metabolism, mutations of the drug target, DNA damage repair, changes in signaling pathways, and evasion of cell death [2]. In these mechanisms, metabolic changes and reactive oxygen species (ROS) levels are non-negligible causes accounting for drug resistance. It alleviates the pressure arising from cancer treatment but also provides new therapeutic targets.

**Figure 1:**
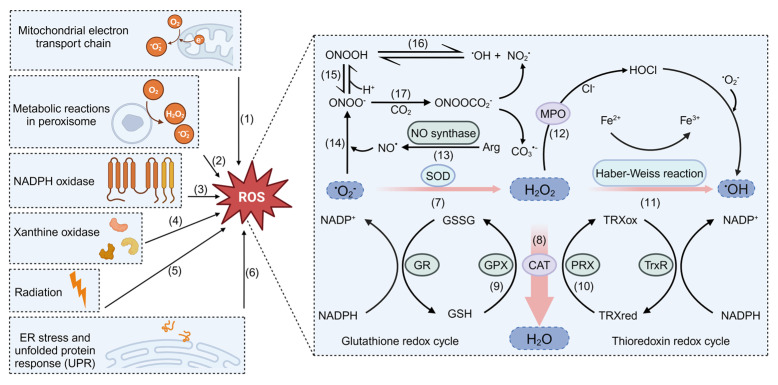
The generation and elimination of ROS and RNS. Cellular ROS derive from various sources; the main source is the mitochondrial electron transport chain (mETC). (1) The oxygen molecules (O_2_) accept electrons in mETC and are converted into superoxide radical anion (O_2_^−^). (2) Like mitochondria, peroxisomes are ubiquitous organelles that produce ROS at high levels through metabolic reactions. (3) And NADPH oxidase (NOX) complex can catalyze the transfer of electrons from NADPH to O_2_ to form O_2_^−^. (4) In addition, xanthine oxidase (XO) has been also regarded as one of the major oxidase enzymes involved in the generation of ROS. (5–6) Some fraction of ROS can also be induced by radiation, endoplasmic reticulum stress, etc. To counteract the damage of ROS accumulation, cells are equipped with antioxidant defense system. (7) First, under the catalysis of superoxide dismutases (SODs), O_2_^−^ can be quickly converted into H_2_O_2_. (8) H_2_O_2_ is then reduced by catalase (CAT), glutathione peroxidase (GPX), or peroxiredoxin (PRX) into H_2_O. (9–10) GPX and PRX utilize the NADPH-induced glutathione (GSH) and reduce thioredoxin (TRXred) as electron donors to maintain redox homeostasis. (11) The H_2_O_2_ can also be converted into hydroxyl radical (·OH) through the iron-dependent Haber–Weiss reaction. (12) Specifically, myeloperoxidase (MPO) catalyzes H_2_O_2_ to generate hypochlorous acid (HOCl), which then reacts with O_2_^−^ to form ·OH. (13) In addition, nitric-oxide synthase (NOS) can catalyze the conversion of L-arginine to L-citrulline and release nitric oxide (NO·). (14–17) NO· is further converted to peroxynitrite (ONOO^−^), peroxynitrous acid (ONOOH), and nitrogen dioxide (·NO_2_), which together form reactive nitrogen species (RNS). GSH, glutathione. GSSG, glutathione disulfide. GR, glutathione reductase. TRXox, oxidized thioredoxin. Cl^−^, chloride ions.

ROS, as byproducts of aerobic metabolism, are a general term for oxygen intermediates with high reactive capacity. In normal cells, the generation and elimination of ROS are strictly regulated to maintain intracellular redox homeostasis (Figure 1). However, to satisfy the substantial materials and energy demands required for rapid proliferation, cancer cells adjust their metabolic network flexibly through a process known as metabolic reprogramming [3]. Metabolic reprogramming increases the synthesis of proteins, nucleotides, and lipids while also triggering the production of ROS [4]. To prevent oxidative damage, cancer cells are equipped with an elaborate antioxidant defense system, including genetic reprogramming of the antioxidant system and metabolic remodeling, which can limit ROS levels below the toxic threshold by increasing reducing equivalents. ROS have also been shown to affect metabolism by serving as a second signal or directly oxidizing the active cysteine residues of various metabolic enzymes [5,6]. The complex interaction between ROS and metabolism can affect drug resistance through multiple mechanisms, including DNA damage repair, cell death pathway, drug metabolism, and so on.

In this review, we summarized the impact of ROS-mediated metabolic abnormalities on tumor drug resistance and highlighted drug sensitization regimens targeting metabolic–redox networks.

## 2. Metabolic–Redox Circuits and Redox Modifications

One hundred years ago, Otto Warburg discovered that tumor cells undergo glycolysis even in the presence of ample oxygen, which opened the prelude of studies about tumor metabolic reprogramming [7]. Hyperactive glycolysis not only provides cells with a very rapid supply of energy but also provides metabolic intermediates for macromolecular biosynthesis [8]. Moreover, metabolic abnormalities or oncogenic activations can disrupt redox homeostasis. Under high metabolic rates, the electrons that leak from the mitochondrial electron transport chain (mETC) react with molecular oxygen to produce superoxide anions (O_2_^−^) and ROS from NADPH oxidases (NOXs), peroxisomes, and endoplasmic reticulum (ER) are also increased [5]. In response to oxidative stress, various antioxidant enzymes such as superoxide dismutases (SODs), catalases (CAT), peroxiredoxins (PRXs), GSH peroxidases (GPXs), and thioredoxins (TRXs) are upregulated or activated to neutralize ROS in tumor cells effectively. Some metabolic enzymes can also exert noncanonical functions to combat excessive ROS. For example, under oxidative stress induced by human papillomavirus, lactate dehydrogenase A (LDH-A) can translocate into the nucleus, where it converts α-ketobutyrate (α-KB) to α-hydroxybutyrate (α-HB). Then, α-HB triggers antioxidant responses mediated by disruptor of telomeric silencing 1-like (DOT1L), promoting the growth of cervical tumors [9]. In addition, ROS also act as a signaling molecule to control tumor metabolism. For instance, elevated levels of ROS inhibit pyruvate kinase 2 (PKM2, the final key enzyme in aerobic glycolysis) acetylation to avoid its lysosomal-dependent degradation, thus weakening the sensitivity of renal cell carcinoma (RCC) to doxorubicin [10].

Apart from indirect regulation, recent studies have found that ROS can directly mediate the oxidative post-translational modifications (OxiPTMs) of metabolic enzymes to finetune the enzyme activity, interaction, and localization, thereby achieving intracellular redox signal transduction. The thiol groups on cysteines in multiple proteins are the main targets for redox modifications [11]. Although cysteine is the least abundant amino acid in proteins, its unique chemistry properties allow it to function as a ‘‘redox switch’’ [12]. Due to the low acid dissociation constant (pKa), cysteine thiols (R-SH) are often dissociated into thiolate anions (R-S^−^) at physiological pH. The thiolate side chains exhibit enhanced nucleophilicity, allowing them to react with oxidants and electrophilic reagents readily. In addition, the low redox potential of cysteine in protein and the presence of positively charged residues contribute to the susceptibility of cysteine to oxidation [13]. Meanwhile, antioxidant enzymes (thioredoxins, glutaredoxins, and peroxiredoxins) can reduce oxidative thiol modifications to maintain intracellular redox homeostasis [14].

Under the nucleophilic attack of ROS, protein thiolate is oxidized to form cysteine sulfenic acid (R-SOH; S-sulfenylation), which is a reversible intermediate. The reversibility of this modification ensures timely transduction of transient signals and immediate defense against oxidative stress to avoid the occurrence of further peroxidation [15]. For example, in the presence of mild oxidative conditions, the Cys215 of protein tyrosine phosphatase-1B (PTP-1B, a critical negative regulator of insulin receptor signaling) was oxidized to sulfenic acid, which promoted the rearrangement of the catalytic site and weakened its phosphatase activity. Reducing agents such as GSH or dithiothreitol (DTT) could ultimately reverse the conformational changes, while excessive H_2_O_2_ treatment led to permanent inactivation of the phosphatase [16,17]. 

The sulfenic acid is generally unstable and rapidly interacts with the surrounding cysteine thiol groups to form intramolecular or intermolecular disulfide bonds (R-S-S-R), or reacts with glutathione (GSH) to form S-glutathionylation (R-SSG). Such redox-active disulfide bonds are generally reversible and can sense changes in intracellular redox potential, thereby affecting the redox balance and metabolism [18]. In addition, glutathionylation can occur not only through the previously mentioned mechanisms but also via direct thiol-disulfide exchanges between protein thiol groups and glutathione disulfide (GSSG) or through reactions between other oxidized derivatives of protein cysteine residues (e.g., thiyl radicals (R-S•) or S-nitrosyls (R-SNO)) and GSH. S-glutathione increases the molecular weight and negative charge of the protein. On the one hand, it can protect the protein from irreversible over-oxidation and permanent inactivation; on the other hand, it can significantly change the conformation and function of the target protein [19,20]. For instance, the glutathionylation of glyceraldehyde-3-phosphate dehydrogenase (GAPDH), a key glycolytic enzyme, leads to its reversible inactivation. This shift diverts the glycolytic flux towards the pentose phosphate pathway, enhancing the production of NADPH to combat oxidative stress [21].

Under the continuous stimulation of oxidative stress or the lack of adjacent thiols, sulfenic acid can be further oxidized to sulfinic acid (R–SO_2_H; S-sulfinylation) or sulfonic acid (R–SO_3_H; S-sulfonylation) [22]. S-sulfinylation and S-sulfonylation have long been considered an irreversible oxidation and of marginal significance in redox signaling. However, the researchers found that sulfiredoxin (SRX) can reduce Prx-SO_2_H to Prx-SH by forming a transient disulfide linkage in the dependence of adenosine 5′ triphosphate (ATP) and magnesium [23,24]. In 2019, Ratcliffe’s team identified an enzyme called cysteamine dioxygenase (ADO) that could use O_2_ as a co-substrate to catalyze the conversion of amino-terminal cysteine of RGS4/5 (regulator of G protein signaling) to cysteine sulfinic acid, thereby regulating the G protein signaling [25]. The discovery of such enzymes challenged the previous understanding that redox modifications are solely nonenzymatic and mediated by free radicals.

**Figure 2:**
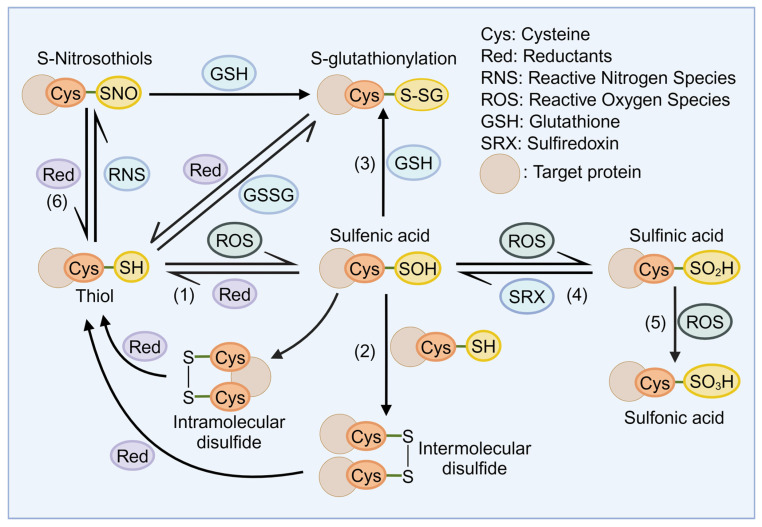
The mechanisms of redox modifications. (1) Under oxidative stress, the thiol groups on cysteines are oxidized to form cysteine sulfenic acid (R-SOH). (2) The sulfenic acid can react with surrounding cysteine thiol groups to form intramolecular or intermolecular disulfide bonds (R–S-S-R), (3) or undergo S-glutathionylation (R–SSG) by binding with glutathione (GSH). (4) The sulfenic acid can also be further oxidized to sulfinic acid (R–SO_2_H) or (5) sulfonic acid (R–SO_3_H). (6) Furthermore, the reactive nitrogen species (RNS) can react with thiols to generate S-nitrosothiols (R-SNOs).

In addition to ROS, the reactive nitrogen species (RNS) can also react with thiols to form S-nitrosylation (R-SNO) [26]. Although S-nitrosylation is unstable, SNO formation at critical cysteine sites can disrupt protein function by affecting protein–molecular interactions, occluding enzyme active centers, and impacting protein oligomerization [27]. As a metabolic enzyme, GAPDH also has numerous moonlighting functions. Studies have shown that nitrosylation of Cys150 on GAPDH by nitric oxide (NO) promoted its binding with Siah1, an E3 ubiquitin ligase. Guided by the nuclear localization signal (NLS) of Siah1, the GAPDH–Siah1 protein complex translocated to the nucleus, where the GAPDH stabilized Siah1 to degrade a variety of nuclear proteins, thus mediating apoptosis [28,29]. The mechanisms of redox modifications are also shown in detail in Figure 2.

Altogether, redox homeostasis and metabolism are dynamic networks that regulate multiple biological processes of tumors, including cell cycle, proliferation, invasion, and metastasis. In the following sections, we will delve into the significant impact of redox homeostasis and metabolism loop on tumor drug resistance.

## 3. Mechanisms of Tumor Drug Resistance Associated with Oxidative Stress

**Figure 3:**
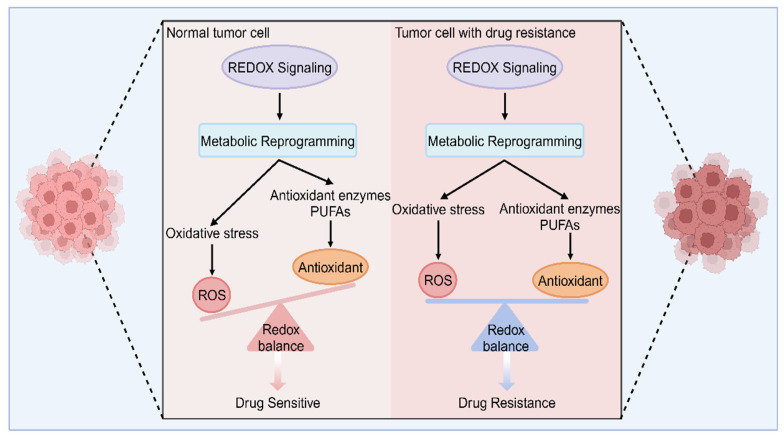
The differences in redox balance between drug sensitivity and drug resistance. Normal tumor cells are sensitive to drugs due to metabolic reprogramming-mediated redox imbalance, excessive oxidative stress, and inadequate antioxidants. In contrast, when oxidation and reduction equivalents are in balance in tumor cells, tumor cells can maintain a redox-stable state through metabolic reprogramming under drug exposure, resulting in drug resistance. PUFAs, polyunsaturated fatty acids.

Vital and effective tumor treatment includes radiotherapy, chemotherapy, targeted therapy, and immunological agents [30,31]. However, almost all types of tumors develop resistance to drugs over time, which diminishes their therapeutic effectiveness or renders them completely ineffective [32,33]. It is widely known that metabolic reprogramming enables tumors to acquire adequate and essential nutrients during their occurrence and development, even under harsh environmental stresses, such as drug treatment [34,35,36]. The activity of many metabolic enzymes will change during metabolic reprogramming, contributing to the dysregulation of signal transduction pathways. In addition, the occurrence, progression, and drug resistance are closely related to redox metabolism. Various antitumor drugs approved by the FDA have been found to rely on increasing ROS production to kill tumor cells [37]. Therefore, figuring out how metabolism influences drug resistance in tumors and how to target the metabolism to reduce or even reverse the drug resistance will be promising for enhancing the effectiveness of cancer therapies. Here, we summarize the mechanisms of drug resistance in tumors and how metabolic–redox circuits influence drug resistance in tumors (Figure 3).

### 3.1. DNA Repair Pathway

In the early stages of tumorigenesis, high levels of ROS can lead to DNA damage and genomic instability [38]. Under oxidative stress, tumor cells can initiate metabolic reprogramming to regulate redox homeostasis and adapt to challenging conditions. Consequently, tumor cells possess a heightened ability to repair DNA and rapidly develop drug resistance under oxidative stress [39]. It has been reported that the overexpression of metallothioneins (MTs) is closely associated with drug resistance [40]. When receiving a highly oxidizing stimulus, an upregulated MTs isoform will protect tumor cells from oxidative toxicity through stabilizing DNA and inhibiting ferroptosis [41]. As a key transcription factor in the cellular antioxidant system, nuclear factor erythroid 2-related factor 2 (Nrf2) effectively alleviates the oxidative stress in tumor cells, thereby enhancing their survival under high oxidative stress exposure [42]. In addition, Nrf2 induced by ROS also can be conducive to facilitating tumor proliferation and enhancing drug resistance to chemotherapy and radiation [43]. Moreover, the mechanism mentioned above also contributes to drug resistance against various DNA-targeting drugs. Notably, once the metabolic imbalance causes ROS accumulation beyond the antioxidant capacity of tumor, it can also damage the DNA of tumor cells and eventually lead to cell death [44,45]. For example, silencing Nrf2 can lead to higher DNA damage with more sensitivity to temozolomide (TMZ) in glioma [42]. Therefore, silencing Nrf2 is considered a strategy for resensitizing tumor cells to drugs that cause DNA damage [46]. 

### 3.2. Cell Death Pathway

In addition to DNA damage, oxidative stress can also target various cell death pathways, including apoptosis, programmed cell necrosis, autophagy, and ferroptosis, leading to two different outcomes: one is the death of tumor cells with powerful oxidative exposure; the other is the evasion of tumor cells for cell death with drug resistance [47].

#### 3.2.1. Apoptosis

A lot of research has verified that apoptosis has two pathways: one is the death receptor pathway and the other is the mitochondrial pathway [48,49]. Intriguingly, oxidative stress is closely associated with both pathways [50,51,52]. Faced with the direct and indirect effects of drugs that induce apoptosis, tumor cells develop a robust antioxidant capacity to enhance their survival [53]. Although moderate levels of ROS are favorable for the occurrence and development of tumor and tumor drug resistance, excessive ROS may sensitize tumors to drugs by inducing apoptosis. Cancer cells are equipped with an elaborated antioxidant system to prevent excessive ROS accumulation. For instance, cyclophilin A (CypA) can mediate a disulfide bond formation between its residues Cys115 and Cys161 when receiving excessive ROS stimulation to resist oxidative stress [54,55,56]. At the same time, PRDX2, as a member of the PRDX family, can form transient mixed-disulfide bonds with CypA so that the oxidizing equivalents of CypA can be transferred to PRDX2. Thus, tumor cells can maintain redox balance and escape from drug-induced apoptosis, contributing to drug resistance. Therefore, the administration of drugs that target CypA, such as cyclosporine A, can enhance the antitumor effect of 5-fluorouracil (5-FU) and oxaliplatin in colorectal cancer (CRC) therapy [54].

#### 3.2.2. Necrosis

Necrosis is a mode of cell death regulated by various signaling pathways, including oxidative-stress-related pathways [57,58]. After receiving the “death signal”, receptor-interacting protein kinase 1 (RIPK1) can be deubiquitinated by cylindromatosis (CYLD) and leads to recruitment of RIPK3 to form a complex and phosphorylate mixed-lineage kinase domain-like (MLKL). Next, RIPK3 phosphorylates the pyruvate dehydrogenase complex (PDC) in mitochondria to promote aerobic respiration and the production of mitochondrial ROS, forming of necrosomes, and eventual programmed cell necrosis [59,60]. Soumya Basu et al. disclosed that potassium-N-(2-hydroxy 3-methoxy-benzaldehyde)-alaninate (PHMBA) exerted an ability to overcome drug resistance through ROS-mediating necrosis. Other research showed that CuPHMBA, as a type of chelate of copper, can also induce cell necrosis based on upregulation of ROS expression to kill tumor cells in both drug-resistant and non-drug-resistant tumor cells [61].

#### 3.2.3. Autophagy 

In previous research, autophagy was often regarded as an antitumor pathway due to its ability to maintain cell homeostasis [62,63]. However, this traditional concept has been challenged with the continuous deepening of research, it is now widely known that autophagy is a double-edged sword during tumor development. Regarded as a restraining force in the development process of tumorigenesis, autophagy can remove damaged mitochondria and suppress ROS accumulation, which is conducive to inhibiting tumors [64]. However, autophagy can also play an inverted role in regulating tumor development by modulating ROS levels. It is observed that autophagy promoted the survival of tumor cells in advanced tumor stages [65]. Moreover, drug resistance may emerge in some cases using therapies targeting autophagy [66]. Zhang et al. found that autophagy participates in cisplatin resistance in ovarian cancer and targeting nucleus accumbens-1 (NAC-1), a regulator involved in autophagy, can further inhibit autophagy and induce oxidative stress to recover the sensitivity of ovarian cancer cells to cisplatin [67].

#### 3.2.4. Ferroptosis 

Ferroptosis is a type of cell death directly caused by metabolic imbalance, which can be influenced by the balance between iron-accumulation-mediated ROS production and the antioxidant system avoiding lipid peroxidation [68,69]. Stearoyl-CoA desaturase-1 (SCD1) is a key enzyme in fatty acid metabolism and various studies have reported that it is closely related to ferroptosis. SCD1 has been regarded as a critical target in the PI3K-Akt-mTOR pathway in drug resistance, which can protect tumor cells from the threat of ferroptosis by promoting monounsaturated fatty acids (MUFA) synthesis [70]. Previous studies have suggested that inhibiting SCD1 expression can sensitize tumor cells to ROS [71,72]. For example, MF-438, as an inhibitor of SCD1, can not only increase ferroptosis and immunogenic cell death (ICD) of tumor cells but also improve the sensibility of tumor cells to radiation therapy [73].

### 3.3. Drug-Related Pathway

In addition to affecting the response of tumor cells to drugs, tumor cells can exhibit drug resistance by influencing the antitumor effect of drugs. For example, tumor cells always develop decreased drug intake and changed drug properties by reducing drug access, increasing drug efflux, and altering drug metabolism to change drug properties and alter drug targets, leading to alleviated antitumor effects of multiple drugs [74,75,76].

#### 3.3.1. Abnormal Drug Transport Systems

Adequate and effective medicine is a strong guarantee of antitumor ability. However, metabolic reprogramming and high levels of ROS in tumor cells often alter drug transport systems so that only a few drugs can stay in the cell to exhibit antitumor effects [77,78]. Therefore, compensating for the loss of drugs to reverse drug resistance is worth studying. Cisplatin is recognized as an effective treatment for ovarian cancer, but its efficacy often is limited by irreversible drug resistance [79,80,81]. It is reported that the expression of tumor necrosis factor receptor-associated protein 1 (TRAP1) will be reduced for strong antioxidant properties to respond to oxidative stress in cisplatin-resistant HGSOC cell lines. During this process, an upregulated P-glycoprotein acted as a drug efflux pump, resulting in drug resistance. This metabolic reprogramming also alters oxidative phosphorylation (OXPHOS), and OXPHOS inhibitor treatment may combat drug resistance [82]. Giorgia Pellavio’s team has reported that oxidative stress may contribute to the upregulated expression of aquaporins 4 (AQP4) and AQP6. AQP4 and AQP6 increase the efflux of H_2_O_2_, resulting in greater tolerance of tumors to drugs that kill tumor cells by increasing ROS in malignant pleural mesothelioma (MPM). Thus, silencing AQPs resensitizes tumor cells to drug-induced hyperoxidation and inhibits the survival and proliferation of tumors [83].

#### 3.3.2. Drug Metabolism Change

Altering drug metabolism to reduce effective forms of drugs is another pathway for drug resistance occurrence. With its elevated levels in various types of tumors, aldehyde dehydrogenase (ALDH) plays a crucial role in catalyzing aldehyde oxidation into carboxylic acids. This process effectively reduces oxidative stress caused by aldehyde accumulation, thereby contributing to drug resistance [84]. 

#### 3.3.3. Drug Target Alteration

Drugs exert antitumor effects by binding to their receptor or protein targets [85]. Complex alteration may occur in tumor cells under oxidative stress and affect many pathways in tumors, especially metabolism-related pathways upon drug exposure. Drug targets may be affected during the alteration and the ability to bind to the target may be lost, resulting in therapy failure. A typical example is epidermal growth factor receptor (EGFR). Researchers have found that EGFR is a crucial receptor tyrosine (Tyr) kinase in tumor survival, growth, and migration due to its phosphorylation and dimerization to activate downstream pathways [86,87]. Thus, researchers have created various EGFR Tyr kinase inhibitors (TKIs), which can specifically bind to EGFR ATP-pocket, leading to the prevention of EGFR phosphorylation and dimerization [88,89,90]. Moreover, its abnormal phosphorylation and dimer structure degradation are closely related to TKI resistance under oxidative stress [91,92,93]. Therefore, applying antioxidants may eliminate excessive ROS and inhibit ROS-mediated drug resistance without abnormal phosphorylation and dimerization [91]. 

### 3.4. Tumor Microenvironment (TME)-Related Pathway

Rather than existing alone, tumor cells coexist with various types of immune cells within the TME [94]. Not only can the cells in TME such as T cells, macrophages, and fibroblasts impact drug resistance, some products, including some types of metabolites in TME, also have an effect on drug resistance. Depending on this dynamic process of mutual influences, both the TME and the cell components may play important roles in regulating tumor drug resistance. For example, fibroblasts in TME have been found to exert a protective effect on tumor cells against drug-mediated apoptosis. Previous studies have reported that fibroblasts can mediate chemotherapy resistance by inhibiting the intracellular accumulation of platinum and increasing intracellular GSH levels [95,96]. Moreover, this drug resistance is also associated with ROS-related NF-κB and TGF-β signaling pathways, which can activate cancer-associated fibroblasts (CAFs) to secrete cytokines, growth factors, and metabolites to promote the development of drug resistance [97]. It is important to note that not all cells within the tumor microenvironment facilitate drug resistance, some cells actually have a suppressive effect on drug resistance. For instance, tumor-infiltrating CD8^+^ T cells have been found to counteract chemotherapy resistance by producing IFN-γ [96]. Notably, ROS can play a significant role in activating T cells and natural killer (NK) cells by recruiting macrophages to eliminate tumor cells [70,95]. Thus, increasing ROS levels may lead to more activated CD8^+^ T cells secreting IFN-γ, potentially reversing drug resistance.

**Figure 4:**
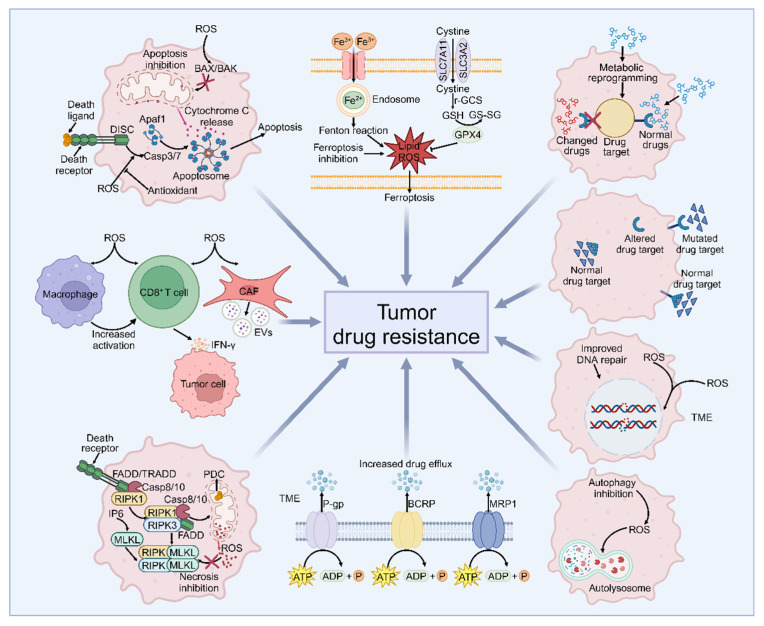
The mechanisms of drug resistance related to redox regulation. Under excessive oxidative-stress-mediated DNA damage, the DNA of tumor cells may remain stable with improved DNA repair ability so that tumor cells become resistant to drugs (Section 3.1). In addition, high levels of ROS may also inhibit apoptosis, necrosis, autophagy, and ferroptosis while improving the antioxidant ability of tumor cells to effectively maintain intracellular redox balance, resulting in drug resistance (Section 3.2). In addition to DNA damage and cell death, tumor cells can also obtain drug resistance by impacting drugs, including inhibiting drug efflux, metabolizing drugs into nonfunctional products, and altering drug targets (Section 3.3). Moreover, TME also plays a significant role in drug resistance. Other cells in TME, such as CD8^+^ T cells and cancer-associated fibroblasts (CAFs), can be influenced by ROS-mediated signaling, contributing to the formation of drug resistance (Section 3.4).

In addition to the mechanisms mentioned earlier, factors such as pyro-death, tumor heterogeneity, and epigenetic inheritance play significant roles [30,98]. These findings have led to the discovery of new targets for drug resistance, paving the way for innovative tumor therapies, including dietary interventions, novel small molecular drugs, drug combinations targeting redox homeostasis, and advanced drug delivery systems. This deeper comprehension of drug resistance mechanisms holds the potential to uncover more drug targets and expand the application of existing therapies (Figure 4).

## 4. Targeting Metabolic–Redox Circuits for Cancer Therapy

According to extensive research, it is widely believed that there are two key strategies for combating tumor drug resistance by targeting redox homeostasis. The first approach involves administering antioxidants to hinder the transformation of normal cells into tumor cells by reducing ROS levels. The second approach entails elevating ROS levels beyond the threshold that tumor cells can withstand, thus causing damage to the tumor cells through various pathways as mentioned earlier [99,100,101]. Based on these two patterns, many drugs have been developed to reverse drug resistance or increase the sensitivity of tumor cells to drugs by targeting redox homeostasis. In the past, various drugs and treatment strategies have been found or developed, including dietary nutrition control, small molecule metabolites, drug combinations, and new drug delivery methods. Here, we summarized some therapeutic strategies that can reduce or reverse resistance.

### 4.1. Dietary Interventions

Given the essential roles of metabolic rewiring and redox signaling in tumor drug resistance, diets or drugs targeting metabolic–redox circuits may become a promising adjunct strategy for sensitizing tumors to anticancer drugs.

#### 4.1.1. Natural Polyphenols

Polyphenols in food and dietary supplements are recognized as natural antioxidants. One classic example is curcumin, a plant polyphenol extracted from the rhizomes of ginger plants, which has been proven to have various physiological activities and low toxicity. Xu’s team reported that curcumin can reverse permeability glycoprotein (P-gp)-mediated multidrug resistance (MDR) at the metabolic level in colorectal cancer. Specifically, treatment with curcumin can reduce the biosynthesis of polyamine by decreasing the expression of ornithine decarboxylase (ODC) and suppressing D-glutamine metabolism. The changes in this metabolic pathway reduced intracellular GSH and ATP levels, which in turn inhibited the antioxidative stress ability and P-gp-mediated drug efflux and eventually reversed the doxorubicin resistance of SW620/Ad300 cells [102]. Epidemiological evidence indicates that diabetes is an independent risk factor for hepatocellular carcinoma (HCC) and is associated with drug resistance to chemotherapy [103]. Curcumin can prevent hyperglycemia-induced intracellular milieu changes and metabolic remodeling, including increased production of ROS and upregulation of metabolic enzymes, thereby exerting the synergistic anticancer effect with doxorubicin [104]. This investigation revealed the potential of curcumin as a dietary supplement in the clinical management of malignancies in diabetic patients. In addition, challenging the traditional view of curcumin as an antioxidant, studies have also reported that it can target and bind to multiple enzymes involved in the ROS metabolic pathway, including carbonyl reductase 1 (CBR1), glutathione-S-transferase phi 1 (GSTP1), aldo-keto reductase family 1 member 1 (AKR1C1), glyoxalase I (GLO1), and so on, resulting in increased ROS levels in leukemia cells and suppressed tumor growth [105]. These studies have revealed the complex and contradictory role of curcumin in regulating ROS and metabolism, which requires further research support.

Neuraminidase 3 (NEU3) is a key enzyme that catalyzes the conversion of ganglioside GM3 to ceramide trihexosides (Gb3); the latter promotes chemotherapy resistance by increasing the expression level of P-gp. Recently, a bound polyphenol from millet bran (BPIS) was reported to inhibit NEU3 expression, thus blocking the catabolism of ganglioside GM3 and improving the sensitivity of drug-resistant colorectal cancer HCT-116/L cells to oxaliplatin (OXA) [106]. Other natural polyphenols, such as resveratrol and polydatin, have also been found to enhance cisplatin-induced oxidative stress in human hepatoma cells via glutamine metabolism inhibition and reduce oxidative stress to fructose-induced hepatic lipid deposition through regulating Keap1/Nrf2 pathway, respectively [107,108]. However, poor bioavailability is also the main obstacle limiting the role of natural polyphenols. Future research focusing on how to effectively improve the absorption and metabolic stability of polyphenols is required [109].

#### 4.1.2. Amino Acid Restriction or Supplementation

Diets restricting specific amino acids, particularly those with differential requirements between normal cells and cancer cells, have emerged as potential therapeutic approaches in various cancer types. 

Glutamine is an energy substrate and carbon source second only to glucose in some cancer cells [110]. Glutamine is also one of the three amino acids needed for GSH synthesis and thus enhances the antioxidant system. Long-term glutamine deprivation can interfere with the redox homeostasis in tumor cells, leading to oxidative stress [111]. Metabolomics analysis showed that, compared with gefitinib-sensitive cell lines, resistant cells A549 showed the upregulation of glutamine synthetase (GS) to promote the utilization of glutamine synthesis, thus protecting cells from gefitinib-induced oxidative stress and death [112]. Current glutamine starvation strategies mainly block the utilization of glutamine by targeting glutaminase (GLS) rather than restricting the intake of glutamine. Telaglenastat (CB-839) is a first-in-class, potent oral inhibitor of GLS, currently being investigated in preclinical trials [113,114]. CB-839 inhibits the endogenous synthesis of GSH by blocking glutamine metabolism, thus significantly weakening the GSH-related antioxidant defense system [115]. Therefore, CB-839 has been evaluated in multiple cancers to synergistically increase the oxidative stress induced by chemotherapeutic drugs such as carfilzomib, 5-fluorouracil, and oxaliplatin and restore chemosensitivity [115,116,117].

Cysteine, as the main target of redox reaction, has a vital function to maintain intracellular redox homeostasis as the limiting substrate for GSH synthesis. The upregulation of lysosomal cysteine transporter MFSD12 promotes lysosomal cysteine storage, which mediates the tolerance of breast cancer cells to anthracyclines [118]. Studies have demonstrated that gastrointestinal cancer patients who received parenteral nutrition containing cysteine had shorter overall survival than those who did not (*p* < 0.001). Xenograft tumor experiments in mice also showed that dietary deprivation of cystine suppressed colon cancer xenograft growth and boosted the antitumor effect of oxaliplatin without noticeable adverse reactions [119]. In conclusion, these studies suggested manipulating cysteine content in nutritional formulations as a potential adjuvant tumor treatment, while the clinical benefits of cysteine deprivation remain to be further investigated. 

In addition to limiting certain amino acids, increasing circulating levels of specific amino acids seems beneficial. Results from two large prospective cohorts show that histidine levels are negatively associated with colorectal cancer risk [120]. For example, dietary histidine supplementation enhances the histidine degradation pathway to consume the tetrahydrofolate (THF) and inhibits nucleotide synthesis. This response enhances the sensitivity of leukemia xenografts to methotrexate, suggesting that histidine supplementation can be an effective candidate for dietary intervention [121].

#### 4.1.3. Vitamins

Dietary micronutrients, such as vitamins, are organic compounds necessary for normal physiological functions. Except for vitamins B3 and D, humans cannot synthesize other vitamins and are highly dependent on external dietary intake [118]. Vitamin supplements as a dietary agent to overcome chemotherapy resistance have been widely confirmed in experiments, in which most data exist for natural antioxidant vitamins A, C, and E.

Vitamin C (L-ascorbic acid) is an essential water-soluble vitamin predominantly present in fruits and vegetables. In KRAS and BRAF mutant colorectal cancer cells, intracellular GSH is consumed during the reduction of oxidized vitamin C (dehydroascorbate, DHA), which causes oxidative stress. The elevated ROS then inactivate GAPDH by promoting the S-glutathionylation of Cys152, thus inhibiting high glycolysis and disturbing energy homeostasis [122]. Similar results are obtained in erlotinib-resistant non-small cell lung cancer cells, suggesting that applying DHA could be a potential strategy against acquired drug resistance [122,123]. In addition, vitamin C can enhance the cytotoxicity of glucose-oxidase-induced H_2_O_2_ and act synergistically with sorafenib in killing HepG2 cells without affecting primary hepatocytes [124]. However, vitamin C may also have a negative impact on anticancer treatments. Vitamin C can directly bind and inactivate bortezomib, a proteasome inhibitor for treating relapsed multiple myeloma [125]. The anticancer mechanism of dietary vitamin C is complicated, and the efficacy and potential interactions of vitamin C with some antineoplastic drugs should be carefully assessed.

Vitamin E, also known as tocopherol, is an essential fat-soluble vitamin that is widely found in vegetable oils, nuts, whole grains, and green leafy vegetables [126]. α-tocopherol is the most widely studied compound among multiple isoforms of vitamin E and has the potential to act as an antioxidant in cancer prevention and treatment. Continuous administration of α-tocopherol to lymphoma-transplanted cancerous mice revealed that α-tocopherol could downregulate the high expression of LDH-A to counteract the Warburg effect [127]. In addition, natural derivatives of vitamin E, such as vitamin E succinate (VES) and D-α-tocopheryl polyethylene glycol succinate (Vitamin E TPGS), have become the present research focus due to their unique structure and biomedical activities [128]. VES is a “mitocans” that interferes with the ubiquinone (UbQ)-binding sites of the mitochondrial complex II, thereby promoting ROS production and blocking ATP synthesis. This in situ ROS magnification of mitochondria promoted the sensitivity of drug-resistant human chronic myelogenous leukemia K562/ADR tumors to doxorubicin hydrochloride (DOX⋅HCl) and provided an effective way to conquer MDR [129]. Because of its excellent safety and biocompatibility, TPGS is widely applied in nanomedicines as an absorption enhancer, emulsifier, and solubilizer to improve the bioavailability of orally administered chemotherapy drugs [130].

As one of the few vitamins that can be synthesized by the human body, numerous epidemiological and experimental data have indicated that vitamin D status is directly associated with cancer risk. Moreover, diverse mechanisms have been gradually revealed to explain its anticancer effects [131,132]. Lisse’s team recently found that, in the vitamin D receptor (VDR)-sensitive MG 63 osteosarcoma cell model, vitamin D treatment can promote the depolarization of the mitochondria membrane to reduce mitochondrial ROS, thereby controlling the growth of osteosarcoma cells [133]. Meanwhile, dietary vitamin D has also been reported to reverse tamoxifen (TAM) resistance in breast cancer cells by inhibiting pro-survival autophagy [134]. Taken together, these results provide evidence for the reversal of drug resistance by dietary vitamin D supplementation through targeting the redox–metabolic circuit.

In addition to the diet manipulation strategies described above, various other elements in the diet, such as minerals and dietary fibers, also have the capacity for cancer prevention [135]. Due to the high feasibility and safety, nutritional interventions have attracted increasing attention. Although there is no substantial evidence that individual nutrients or types of foods can protect the human body from cancer, the proper combination of dietary patterns that specifically target the metabolic vulnerability of tumors can reduce the risk of cancer. 

### 4.2. Novel Chemotherapy and Targeted Therapy Agents

In addition to dietary interventions, chemosynthetic drugs remain the primary strategies for tumor treatment. For example, chemotherapy serves as the footstone treatment for triple-negative breast cancer (TNBC), with doxorubicin (DOX) being the most frequently used drug. However, its effectiveness is often compromised by drug resistance [136,137]. To overcome this dilemma, Chen et al. screened for proteins associated with DOX resistance and finally found thioredoxin-interacting protein (TXNIP). TXNIP exhibited a different expression between drug-resistant and non-drug-resistant tumor cells, suggesting a potential role in drug resistance. Further study implied that TXNIP can increase ROS levels and cause DNA damage, leading to the apoptosis of DOX-resistant tumor cells. In addition, according to previous reports, c-Myc can negatively regulate TXNIP. Thus, 10058-F4, an inhibitor of c-Myc, can promote TXNIP expression, thereby increasing ROS production and reducing DOX drug resistance [138,139,140]. A novel TKI named APG-2449 has been found to exhibit inhibitory activity for anaplastic lymphoma kinase (ALK), ROS proto-oncogene 1 receptor tyrosine kinase (ROS1), and focal adhesion kinase (FAK). Because of its triple-kinase inhibitor capabilities, APG-2449 can play a persistent antitumor role in tumor cells with acquired (secondary) ALK- and ROS1-resistant mutations and suppress the FAK signaling pathway to reawaken the sensitivity of ovarian cancer cells to chemotherapy [141]. Sorafenib is a chemotherapy drug commonly used in patients with advanced HCC. Unfortunately, the emergence of drug resistance often limits its effectiveness. Research indicates that ROS plays a key role in sorafenib resistance [142,143]. Chen et al. extracted data from GEO microarray data (GSE94550) and then found that the expression of SOD1 reduced, while toll-like receptor (TLR)-9 and Beclin-1 are overexpressed in sorafenib-resistant huh7 cells. Besides this, they also found that patients with overexpression of TLR-9 often have shorter overall survival compared to those with normal TLR-9 expression. This suggests that TLR-9 overexpression may contribute to sorafenib resistance by decreasing SOD1 expression and increasing oxidative stress [144]. Thus, using an inhibitor of TLR-9 (such as ODN2088 and ODN TTAGGG (A151)) may have an inhibitory effect on sorafenib resistance. Unfortunately, there is few research on the inhibition of TLR-9 by ODN2088 and ODN TTAGGG (A151) leading to the reversal of drug resistance. Therefore, identifying additional targets associated with drug resistance and developing more targeted therapies are crucial for restoring the efficacy of existing anticancer drugs.

### 4.3. Drug Combinations Targeting Redox Homeostasis

Besides applying a drug alone for treatment, drug combination has emerged as an attractive method for tumors with drug resistance and achieved surprising therapeutic effects. Wu et al. found that co-exposure of vitamin C and the oxidizing drug arsenic trioxide (ATO) induced cell toxicity by promoting the generation of ROS. Compared to ATO treatment alone, a drug combination of vitamin C and ATO can lead to enhanced apoptosis and increased sensibility in response to oxidative stress [145]. As an inhibitor of STAT3, stattic can effectively improve the proapoptotic effect of gemcitabine and make pancreatic cancer cells more sensitive to gemcitabine, which may be induced by the Nrf2/HO-1 signal inactivation-mediated oxidative stress [146]. Similarly, the Nrf2/HO-1 signal pathway is also a critical axis for tumor cells to tolerate drug-induced oxidative stress under the treatment of Cabozantinib, which has been applied to advanced renal cell carcinoma. Rawat reports that combinational use of Cabozantinib and Honokiol can increase ROS by suppressing antioxidant pathways in tumor cells, contributing to enhanced oxidative stress and increased tumor cell death [147]. Sharma et al. found that ROS generation was increased in regorafenib-resistant HCT116 and HT29 cells under the combination treatment of regorafenib and ruthenium complex, leading to induced ERK phosphorylation and increased subsequent apoptosis. In addition, regorafenib-resistant tumor cells also exhibit more sensitivity to ruthenium complex due to downregulating the expression and phosphorylation of Akt, which is closely associated with the survival of tumor cells [148]. 

### 4.4. Novel Drug Delivery Systems

Improving the efficacy of drugs and increasing drug retention are promising methods for tumor treatments. Nanoparticulate drug delivery systems (Nano-DDSs) have been regarded as a tool with longer drug retention time and more specific target affinity, which play a critical role in antitumor effects [149]. Nano-DDS is also involved in targeting redox homeostasis to reverse resistance. Chen et al. synthesized PEG-PPS-GSNO nanoparticles that were sensitive to oxidative stress. Delivering DOX with the PEG-PPS-GSNO nanoparticles exhibited an antitumor role through releasing DOX and NO, respectively, resulting in DOX accumulation and resistance reversing in tumor cells through increasing ROS [150]. Other research has combined nanotechnology with phototherapy to deal with drug resistance by developing a PTD/TT/IR780 nanoparticle that was composed of PTD (ROS-triggered doxorubicin prodrug), TT (mitochondrial-targeted D-α-tocopherol polyethylene glycol succinate (TPP-TPGS)), and IR780. Among nanoparticle components, PTD is a prodrug that is sensitive to ROS, TT is an inhibitor of P-glycoprotein, and IR780 can induce ROS generation in the presence of an 808 nm near-infrared laser. Applying this nanoparticle may suppress the function of P-glycoponents, which leads to the reduction in drug efflux and promotes tumor cell apoptosis via increasing ROS production [151] (Table 1).

## 5. Discussion

Although various targeted metabolic–redox nexus therapies have been developed and gradually applied to clinical treatment, many unresolved issues remain. Due to the instability and reversibility of redox modifications, it is incredibly vulnerable to external oxygen to cause non-physiological oxidation. The current techniques to assay cysteine redox regulation focus on chemical-labeling technology and mass spectrometry (MS)-based redox proteomics [165]. However, these detection methods have problems, with complicated steps, difficulty detecting non-surface cysteines, and lack of spatial-temporal specificity, which also limits the development of redox modification research and redox-targeted therapy. Therefore, it is rational to infer that the direct redox modifications have not been thoroughly explored in previous research on regulating metabolic enzymes by ROS, and more practical and efficient technology is needed.

Since redox and metabolic networks are flexible and plastic, targeted therapy may lead to rapid redox and metabolic adaptations, rendering cancer cells resistant. Intertumoral and intratumoral redox and metabolic heterogeneity also limit the application of targeted drugs [166]. Developing more precise protocols to identify and track targets for individualized treatment rather than homogenous treatment will be a promising research direction.

Furthermore, the contradictory effect of ROS in tumor cells also implies the possible adverse effects of antioxidant therapy. Long-term supplementation with the antioxidants N-acetylcysteine and vitamin E could stabilize the transcription factor BACH1 by reducing levels of ROS and free heme. BACH1 activates the transcription of glycolytic enzymes hexokinase 2 (HK2) and GAPDH, thus increasing glucose uptake, glycolysis rates, and lactate secretion to promote KRAS-driven lung cancer metastasis [167]. In addition, polyphenol and gallic acid have also been reported to restore the TCF4–chromatin association and the hyperactivation of WNT in gut-sterilized p53-mutant mice, thus promoting intestinal malignant phenotypes [168]. These studies have refreshed our understanding of antioxidants, and there may be an urgent need to re-examine and reposition the anticancer effect of antioxidants.

## Figures and Tables

**Table 1:** Small molecular drugs for reversing drug resistance by target metabolism and ROS.

Drug	Cancer Cell Type	Effective Concentration	Research Model	Administration	Outcome/Result/Prognosis	Mechanisms	Reference
Curcumin	Colon cancer	5.5 μM	SW620 and SW620/Ad300 cells	In vitro	Cur dramatically enhanced the Dox-induced early apoptosis and late apoptosis in Dox-resistant SW620/Ad300 cells with the co-administration of Dox. The apoptosis in SW620/Ad300 cells were 4.60% and 17.47% in Dox group and Cur + Dox group, respectively.	Curcumin inhibits the biosynthesis of polyamine by decreasing the expression of ornithine decarboxylase (ODC). It suppresses D-glutamine metabolism, decreasing the anti-oxidative stress ability and eventually reversing doxorubicin resistance.	[102]
	Hepatocellular carcinoma	5 μM	Hep G2 cells	In vitro	5 or 10 µM concentration of curcumin treatment was able to resist the extracellular PH and lactic acid changes of Hep G2 cells in high glucose medium, and exerted synergistic effects with doxorubicin.	Curcumin inhibits the elevated expression of metabolic enzymes and diminishes ROS production against high glucose-induced chemoresistance.	[104]
	Chronic myeloid leukemia	25 mg/kg (i.p.) 50 and 75 μM (in vitro)	Xenograft model (PDTX) derived from leukemia patients, K562 cells	In vivo and in vitro	Curcumin suppresses tumor formation in vivo and induces irreversible growth inhibition in vitro.	Curcumin specifically inhibits tumor growth by increasing ROS levels over the threshold through inhibiting a series of enzymes (carbonyl reductase, glutathione-S-transferase, glyoxalase, etc.)	[105]
Triptolide (TPL)	Different types of cancer	30 nM	IDH1-mutated BTIC TS603 cells	In vitro	Triptolide reduced cellular proliferation by 75% in IDH1-mutated cells. IDH1-mutated cells were more vulnerable to triptolide treatment, with an IC50 of 15 nM, as opposed to an IC50 of 60 nM for IDH1 wild-type cells, indicating that triptolide exhibited stronger cytotoxicity for IDH1-mutated cells.	Triptolide induces oxidative damage by reducing Nrf2-driven glutathione metabolism, thus suppressing IDH1-mutated malignancy.	[152]
Deoxyelephantopin (DET)	Melanoma	20 mg/kg	A375 cells	In vivo	Xenograft A375 tumor masses in NOD/SCID mice were inhibited by PLX4032 (20 mg/kg/day, 24 doses in total), DET and DETD-35 (20 mg/kg/every 2 days, 12 doses in total) 71.9%, 47.5% and 70.5%, respectively. DET−PLX4032 and DETD-35 −PLX4032 combination as an adjuvant therapy inhibited 42.0% and 65.2%, respectively of A375-R tumor mass in mice, indicating a synergistic action of DETD-35 and the BRAFi drug.	DET mediates susceptibility to vemurafenib by triggering the accumulation of lipid ROS and regulating the expression of cytosolic phospholipase A2 to reprogram fatty acid metabolism	[153]
Quercetin (Que)	Colon cancer	33 μM	SW620/Ad300 cells	In vitro	Que significantly improved the cytotoxicity of Dox in SW620/Ad300 cells (3.66 ± 0.0024 μM), compared to that in Que-untreated SW620/Ad300 cells and reversed P-gp-mediated MDR in SW620/Ad300 cells.	Quercetin down-regulates the glutamine transporter SLC1A5 to block glutamine metabolism and promote the increased intracellular accumulation of doxorubicin	[154]
Resveratrol (RV)	Hepatocellular carcinoma	12.5 μg/mL	C3A and SMCC7721 cells	In vitro	12.5 μg/mL RV enhances 0.625 μg/mL CDDP induced apoptosis in C3A and SMCC7721 cells	Resveratrol decreases the absorption of glutamine by reducing the expression of glutamine transporter ASCT2 and increases ROS production to enhance cisplatin-induced apoptosis.	[107]
Metformin	Skin squamous cell carcinoma	25-150 μM	SCC13 and A431 cells	In vitro	The combined treatment of metformin (25–150 μM) and PDT (0.3 mM MAL, 5 h, and 23 J/cm^2^ in SCC13 cell line and 7 J/cm^2^ in A431 cell lines) significantly reduced the cell survival rate of SCC13 and A431, and had a great cytotoxic effect on 3D cultures.	Metformin modulates energetic metabolism and increases ROS generation, sensitizing to photodynamic therapy (PDT).	[155]
Epalrestat	Lung cancer	50 mg/kg	HCC827-CDX tumors implanted subcutaneously in BALB/c nu/nu mice	In vivo	The combination treatment of epalrestat and gefitinib effectively retarded tumor growth, reduced tumor volumes, and even blocked relapse.	AKR1B1 inhibitor epalrestat can down-regulate the SLC7A11, thereby inhibiting cystine uptake, glutathione de novo synthesis, and ROS scavenging to overcome resistance to EGFR TKIs.	[156]
Dichloroacetate (DCA)	Breast Cancer	60 mM	EMT6 and 4T1 cells	In vitro	60 mM DCA significantly (*p* < 0.05) overcame hypoxic radioresistance with enhancement ratios of 2.3 and 1.5 at 60 mM for EMT6 and 4T1 tumor cells, respectively	Dichloroacetate shifts glycolysis-to-OXPHOS metabolism by decreasing phosphorylated pyruvate dehydrogenase (PDH) and significantly increases ROS production to radiosensitize hypoxic breast cancer cells.	[157]
2-Deoxy-D-glucose (2-DG)	Glioblastoma	The IC_10_, IC_25_ and IC_50_ doses of SF126 cells (1.25, 6.5 and 35.25 mM, respectively) and SF763 cells (0.55, 5.5 and 56.85 mM, respectively)	BCNU-resistant SF126 and SF763 cells	In vitro	Compared with BCNU alone treated groups, the cell survival rates of groups pretreated with the IC_10_, IC_25_ and IC_50_ doses of 2-DG for 5 h followed by exposure to BCNU for 24 h decreased significantly.	2-DG can overcome the resistance of glioblastoma cells to chloroethyl nitrosourea (CENUs) by inhibiting glycolysis, increasing oxidative stress, and endoplasmic reticulum stress in tumor cells.	[158]
3-mercaptopropionic acid	Melanoma	6.37 μM	Vemurafenib -resistant melanoma A2058R cells	In vitro	The IC_50_ of 3-mercaptopicolinic acid combined with vemurafenib was significantly lower than that of vemurafenib alone, which indicated that 3-mercaptopicolinic acid increased the resistance inhibition of vemurafenib	3-mercaptopropionic acid sensitizes vemurafenib by selectively inhibiting phosphoenolpyruvate-calorie kinase 1(PCK1), and suppresses the resistance via the Akt/PCK1/ROS axis.	[159]
AZD3965	Small cell lung cancer	100 mg/kg	Mice bearing H526 tumours	In vivo	Administration of AZD3965 alone for seven days increased the time for tumors to reach 1000 mm^3^ from 8 to 12 days; for radiation alone this time was 18 days, which was increased to 25 days when combined with AZD3965.	AZD3965 reduces bidirectional lactate transport and increases oxidative stress, which in turn enhances radiosensitivity.	[160]
Catechin (CA)	Gastric cancer	10 μM	SNU620, SNU620/5FU, AGS, and RKO cells	In vitro	The combination treatment of CA and 5FU significantly reduced the viability of cells, compared to a single treatment of 5FU in glycolytic cells including AGS and RKO.	Catechin can act as a suppressor of LDHA expression, thereby inducing mitochondrial ROS-mediated apoptosis in 5FU-resistant cells.	[161]
Cyclosporine A (CsA)	Colorectal cancer	2.5 μM	HCT116 cells, BALB/c nude mice are subcutaneously injected with LoVo/OXAR cells model.	In vivo and in vitro	CsA enhanced the efficacy of 5-FU and OXA in CRC cells. Analysis of the tumor size, tumor growth rate, and tumor weight revealed that CsA synergized with OXA in CRC treatment.	The target of cyclosporine A is CypA, which can reduce ROS production to maintain redox balance by forming an intramolecular disulfide bond between Cys115 and Cys161 under oxidative stress.	[54]
Dihydroartemisinin (DHA)	Colorectal cancer	4 g/kg	Transgenic model of intestinal cancer, driven by either Apc mutation, or combined Apc and Kras (G12D) mutations.	In vivo	After be treating daily with high-dose vitamin C (IP, 4 g/kg) for 5–7 weeks, the Apc^flox/flox^ mice showed no difference in polyp burden, and Apc^flox/flox^/Kras^G12D^ mice had significantly fewer and smaller small intestine polyps (76 vs. 165 in control group).	DHA leads to oxidative stress to inactivate GAPDH, thus mediating energetic crisis and cell death of KRAS or BRAF mutant cells.	[122]
	Leukemia	40.54 ± 1.75 μM	Drug-resistant K562/ADM leukemia cells	In vitro	Compared with no treatment, DHA treatment significantly reduced the viability of both cell lines in a dose- and time-dependent manner. Moreover, the cytotoxicity of ADM was increased following treatment with DHA in MDR K562/ADM cells	DHA enhances the sensitivity of leukemia cells to ademycin by decreasing GSH levels and up-regulating ROS levels.	[162]
Manganese (III) meso-tetrakis N-ethylpyridinium-2-yl porphyrin (MnTE-2-PyP^5+^)	Lymphoma	50 nM	WEHI7.2 cells	In vitro	In combination with 2DG, MnTE-2-PyP5+ decreased the cellular ATP levels, more than 2DG treatment alone. In combination with 2DG, MnTE-2-PyP^5+^ enhanced the ability of 2DG to induce cell death by 14.65 ± 1.6%	MnTE-2-PyP^5+^ increased dexamethasone-induced mitochondrial ROS and oxidation of the mitochondrial glutathione pool in lymphoma cells.	[163]
Potassium-N-(2-hydroxy-3-methoxy-benzaldehyde)-alaninate (PHMBA)	Erlich ascites carcinoma (EAC)	0.09 ± 0.01 mM	CCRF-CEM, CEM/ADR5000, EAC/S, and EAC/Dox cells	In vitro	PHMBA-induced cytotoxic effects on CCRF-CEM and CEM/ADR5000 cell lines in a dose and time-dependent manner and also decreased the cell viability of EAC/S and EAC/Dox cells.	PHMBA can overcome drug resistance and eliminate both doxorubicin-resistant and -sensitive T lymphoblastic leukemia cells and Ehrlich ascites carcinoma (EAC) cells through oxidative stress-mediated mitochondrial pathway	[164]
10058-F4	Triple-negative breast cancer (TNBC)	20 mg/kg	Xenograft mouse model	In vivo	The tumor growth rate of nude mice treated with the combination of 10058-F4 and DOX significantly slowed down from the 12 day of treatment. At the end of the drug treatment, the tumor size and weight of the mice receiving the combination therapy were significantly lower than those of the other groups, indicating the effectiveness of the drug combination.	As a c-Myc inhibitor, 10058-F4 can improve the expression of TXNIP, which exerts the effect of increasing ROS generation, resulting in reduced drug resistance.	[138]
APG-2449	Ovarian cancer	50, 100, and 150 mg/kg	*ALK* or *ROS1* murine xenograft tumor models	In vivo	Compared to each single agent, the combination of APG-2449 and paclitaxel enhanced antitumor activity in all 6 PDX models. In addition, the combination significantly augmented antitumor activity in the PDX models, with a synergy ratio greater than 2.	APG-2449 can suppress ALK and ROS1 so that it can exert anti-tumor effects in ALK- and ROS1-resistant tumor cells and can inhibit the FAK signaling pathway to reawaken the sensitivity of ovarian cancer cells to chemotherapy	[141]
Hydroxychloroquine (HCQ)	Hepatocellular carcinoma	12.69~13.6 μM, 30 mg/kg	HCC-resistant cell lines (Huh7-SR and HepG2-SR), xenograft mouse Huh7-SR tumor model	In vivo and in vitro	The combined effect of HCQ with sorafenib treatment synergistically inhibited and re-sensitized HCC-resistant cell proliferation to sorafenib. And the tumor volume over time clearly showed that HCQ treatment together with sorafenib combination resulted in significantly delayed tumorigenesis, while the vehicle and HCQ and sorafenib alone groups displayed no significant modulation of tumor volume and weight.	Hydroxychloroquine (HCQ) can inhibit the expression of TLR-9 and reverse resistance by targeting TLR-9 to break its protection for SOD1 that is overexpressed in sorafenib-resistant huh7 cells.	[144]

“Drug” represents various drugs with reversing drug resistance effects in tumor cells; “Cancer cell type” means which type of cancer cells have been chosen to study the effects and mechanisms of these drugs; “Effective concentration” means the concentration dose of the drug used in the study; “Research model” means what type of model is used in this research, such as cell experiments and animal experiments; “Administration” means the delivery of this drug including in vitro and in vivo; “outcome/result/prognosis” means the outcome/result/prognosis of this study under corresponding conditions and “Mechanisms” means how these drugs reverse drug resistance.
